# Systematic Review of the Key Factors Influencing the Indoor Airborne Spread of SARS-CoV-2

**DOI:** 10.3390/pathogens12030382

**Published:** 2023-02-27

**Authors:** Simon de Crane D’Heysselaer, Gianni Parisi, Maxime Lisson, Olivier Bruyère, Anne-Françoise Donneau, Sebastien Fontaine, Laurent Gillet, Fabrice Bureau, Gilles Darcis, Etienne Thiry, Mariette Ducatez, Chantal J. Snoeck, Stéphan Zientara, Nadia Haddad, Marie-France Humblet, Louisa F. Ludwig-Begall, Georges Daube, Damien Thiry, Benoît Misset, Bernard Lambermont, Yacine Tandjaoui-Lambiotte, Jean-Raph Zahar, Kevin Sartor, Catherine Noël, Claude Saegerman, Eric Haubruge

**Affiliations:** 1 TERRA Research Centre, Gembloux Agro-Bio Tech, University of Liège, 5030 Gembloux, Belgium; 2 Research Unit in Epidemiology and Risk Analysis Applied to Veterinary Sciences (UREAR-ULiege), FARAH Research Centre, Faculty of Veterinary Medicine, University of Liege, 4000 Liege, Belgium; 3 Division of Public Health, Epidemiology and Health Economics, Faculty of Medicine, University of Liège, 4000 Liège, Belgium; 4 Biostatistics Unit, Faculty of Medicine, University of Liège, 4000 Liège, Belgium; 5 Institute for Research in Social Sciences (IRSS), Faculty of Social Sciences, University of Liege, 4000 Liège, Belgium; 6 Immunology-Vaccinology Laboratory, FARAH Research Center, Faculty of Veterinary Medicine, University of Liège, 4000 Liège, Belgium; 7 Laboratory of Cellular and Molecular Immunology, GIGA Institute, University of Liege, 4000 Liège, Belgium; 8 Infectious Diseases Department, Centre Hospitalier Universitaire de Liège, 4000 Liège, Belgium; 9 Veterinary Virology and Animal Viral Diseases, FARAH Research Centre, Department of Infectious and Parasitic Diseases, Faculty of Veterinary Medicine, University of Liège, 4000 Liège, Belgium; 10 IHAP, Université de Toulouse, INRAE, ENVT, 31000 Toulouse, France; 11 Clinical and Applied Virology Group, Department of Infection and Immunity, Luxembourg Institute of Health, 4354 Esch-sur-Alzette, Luxembourg; 12 UMR1161 Virologie, INRAE, Ecole Nationale Vétérinaire d’Alfort, Anses, Université Paris-Est, F-94700 Maisons-Alfort, France; 13 UMR BIPAR 956, Anses, INRAE, Ecole Nationale Vétérinaire d’Alfort, Université Paris-Est, 94700 Maisons-Alfort, France; 14 Department of Occupational Safety and Health, University of Liege, 4000 Liege, Belgium; 15 Laboratoire de Microbiologie des Denrées Alimentaires, FARAH Research Center, Faculty of Veterinary Medicine, University of Liège, 4000 Liège, Belgium; 16 Bacteriology, FARAH Research Center, Faculty of Veterinary Medicine, University of Liege, 4000 Liège, Belgium; 17 Service des Soins Intensifs, CHU Sart Tilman, Department des Sciences Cliniques, University of Liège, 4000 Liege, Belgium; 18 Laboratoire Hypoxie and Poumon INSERM U1272, Service de Réanimation Médico-Chirurgicale, CHU Avicenne, Assistance Publique-Hôpitaux de Paris, 93000 Bobigny, France; 19 CHU Avicenne-APHP, 93000 Bobigny, France; 20 Planification: Energie—Environnement, Département d’Aérospatiale et Mécanique, Systèmes Énergétiques, University of Liège, 4000 Liège, Belgium

**Keywords:** SARS-CoV-2, COVID-19, airborne transmission, indoor, mitigation measures, CO_2_, air quality

## Abstract

The COVID-19 pandemic due to the Severe Acute Respiratory Syndrome Coronavirus 2 (SARS-CoV-2) has been plaguing the world since late 2019/early 2020 and has changed the way we function as a society, halting both economic and social activities worldwide. Classrooms, offices, restaurants, public transport, and other enclosed spaces that typically gather large groups of people indoors, and are considered focal points for the spread of the virus. For society to be able to go “back to normal”, it is crucial to keep these places open and functioning. An understanding of the transmission modes occurring in these contexts is essential to set up effective infection control strategies. This understanding was made using a systematic review, according to the Preferred Reporting Items for Systematic reviews and Meta-Analyses statement (PRISMA) 2020 guidelines. We analyze the different parameters influencing airborne transmission indoors, the mathematical models proposed to understand it, and discuss how we can act on these parameters. Methods to judge infection risks through the analysis of the indoor air quality are described. Various mitigation measures are listed, and their efficiency, feasibility, and acceptability are ranked by a panel of experts in the field. Thus, effective ventilation procedures controlled by CO_2_-monitoring, continued mask wearing, and a strategic control of room occupancy, among other measures, are put forth to enable a safe return to these essential places.

## 1. Introduction

In recent decades, various members of the coronavirus family have been associated with outbreaks of respiratory diseases. Notable examples are the Severe Acute Respiratory Syndrome (SARS) outbreak in 2002, and the Middle East Respiratory Syndrome (MERS) outbreak in 2013. The latest outbreak finds its origins in Wuhan, China, where cases of unexplained pneumonia were found in December 2019. On 30 January 2020, the World Health Organization (WHO) issued a Public Health Emergency of International Concern following quick efforts to isolate the causative agent of Coronavirus Disease 2019 (COVID-19). These efforts identified it as an RNA virus from the Coronaviridae family, and showed significant similarities between this novel virus, and the one responsible for the 2002 SARS outbreak, namely SARS-CoV-1. Thus, the novel virus was called Severe Acute Respiratory Syndrome Coronavirus 2 (SARS-CoV-2). This virus rapidly spread around the world, and on 11 March 2020, the WHO declared a global pandemic [1]. At the end of September 2022, more than 2.5 years after the first identification of the virus, the WHO counted over 614 million confirmed COVID-19 cases worldwide, including over 6.5 million deaths [2].

Acknowledging the threat that was posed by this pandemic, the global scientific community acted rapidly on the development of a vaccine. Various private developers, but also projects led by academic or public initiatives, gave rise to large-scale efforts to rapidly develop a vaccine against COVID-19. The publication of the genetic sequence of SARS-CoV-2 on 11 January 2020 started this vast research and development activity [3]. Today, different vaccines have been developed and are still being deployed worldwide. These vaccines have been distributed worldwide, albeit inequitably; over half of the early doses produced in November 2020 went to high-earning countries, leaving developing countries trailing far behind [4]. Vaccination strategies are well underway, and at the end of September 2022, more than 12.6 billion vaccine doses were administered, with most of the high-earning countries presenting full-vaccination (requiring 2 doses) numbers of over 75% of the whole population [2,5].

However, partly due the development of new variants of the virus, the different vaccines have not been able to completely root out the virus. This means that besides vaccination strategies, several additional measures still need to be put in place to contain the spread of the virus. This is especially important as the different countries and populations that have been hit by this pandemic are urgently seeking a way to go “back to normal”. However, places traditionally known to accept large gatherings of people indoors, such as schools, offices, restaurants, and public transports present larger infection risks. Epidemiologists agree that the SARS-CoV-2 virus could stay present in the longer term and present seasonal peaks, in the same way as other respiratory viral infections, such as the influenza virus [6]. We will thus have to find a way to cope with the presence of the virus while maintaining those indoor activities that are essential for the correct functioning of our modern-day society.

Various transmission routes of the SARS-CoV-2 virus have already been identified. First, direct transmission occurs when respiratory droplets coming from an infected individual are inhaled by a susceptible individual at close range [7,8]. This transmission mode can occur at distances under 3 m, and was considered the only mode of SARS-CoV-2 airborne transmission by the WHO at the start of the pandemic in 2020. Second, indirect transmission occurs when a surface is contaminated via viral particles produced by an infected individual. These infectious surfaces (fomites) can then, in turn, transmit the virus when touched. Finally, the indirect airborne transmission mode, occurring through the inhalation of smaller suspended respiratory droplets at farther distances, was widely debated in the first stages of the pandemic, but is now largely accepted and even considered as the main transmission route [7,8,9,10,11]. When considering indoor environments, this transmission mode is particularly significant, and can occur at longer distances, as the infectious droplets stay suspended and can travel following air flows and currents [12]. In addition, various super-spreading events have been documented, during which the first two modes of transmission do not suffice to explain the mechanisms of infection at these events [13,14,15]. The initial 2 m distancing rule recommended by the WHO is useful to protect against the first direct mode of transmission, but is not sufficient to prevent infection through suspended particles at longer distances [16,17].

The exact terms used when discussing airborne transmission are not precisely defined. Indeed, definitions may differ when originating from medical scientists, epidemiologists, chemists or physicists, and the interpretation by the general public may again be completely different. Therefore, a quick definition of the commonly used terms to describe these transmission modes is necessary, and available in Table 1.

The aim of this systematic review is to identify the different mechanisms active in the infection risk assessment of indoor spaces. The comprehension of the different parameters that can influence this risk is crucial for a better understanding of the infection mechanisms. Based on this, it should be possible to propose appropriate risk mitigation measures. These measures should be applicable all year long, or different measures should be proposed to counteract the higher risk in winter settings.

## 2. Materials and Methods

This systematic review was performed according to the Preferred Reporting Items for Systematic reviews and Meta-Analyses statement (PRISMA) guidelines [22], and based on publications in English retrieved on PubMed and Scopus databases. The search was conducted using the following search strings:

- ((SARS-CoV-2) and (COVID-19)) and ((Indoor) or (Inside)) and ((CO_2_) or (carbon dioxide))
- ((SARS-CoV-2) and (COVID-19)) and ((Indoor) or (Inside)) and ((airborne transmission) or (aerosol transmission))
- ((SARS-CoV-2) and (COVID-19)) and ((hvac) or (air quality control) or (air conditioning))
- ((SARS-CoV-2) and (COVID-19)) and ((Indoor) or (Inside)) and ((Temperature) or (Humidity))
- ((SARS-CoV-2) and (COVID-19)) and ((Indoor) or (Inside)) and ((Fine particles) or (Fine Particulate matter) or (PM))
- ((SARS-CoV-2) and (COVID-19)) and ((Indoor) or (Inside)) and ((aerosol) or (bioaerosol) or (airborne))
- ((SARS-CoV-2) and (COVID-19)) and ((Indoor) or (Inside)) and (air) and ((mitigation control) or (mitigation measures) or (mitigation))

While screening the records, different inclusion and exclusion criteria were taken into account. Publications that treated the production of bioaerosols in specific medical settings, such as chirurgical interventions or dentistry activities, where strict measures are needed, were considered out of the scope of this review. The same applied to publications with either a strong focus on novel mask technologies, or reference to outdoor environmental aspects. Publications pertaining to transmission in the context of public transport (e.g., buses, airplanes, and trains) were also excluded, as these spaces present very different characteristics compared to “fixed and stable” indoor environments. Only records produced in English and with full-text availability were selected.

## 3. Results and Discussion

### 3.1. Selection of Publications Related to the Indoor Airborne Spread of SARS-CoV-2

**Figure 1:**
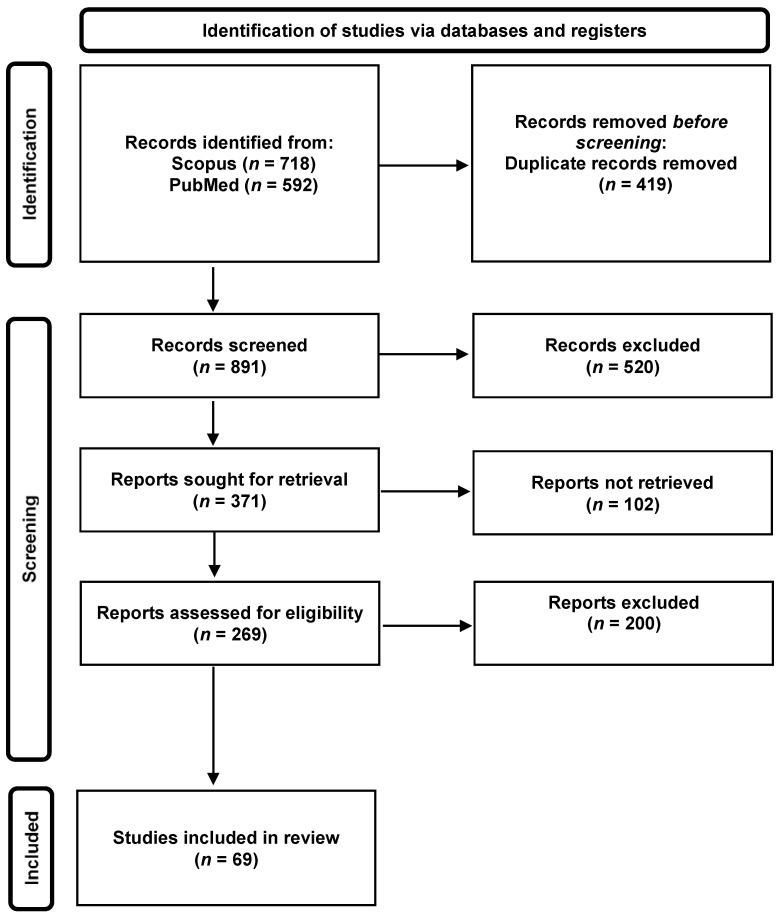
Flowchart representing the record selection process according to the PRISMA guidelines [22].

The selection and exclusion process of the different retrieved studies is presented in Figure 1. The PRISMA 2020 Checklist is presented in Appendix A.

**Figure 2:**
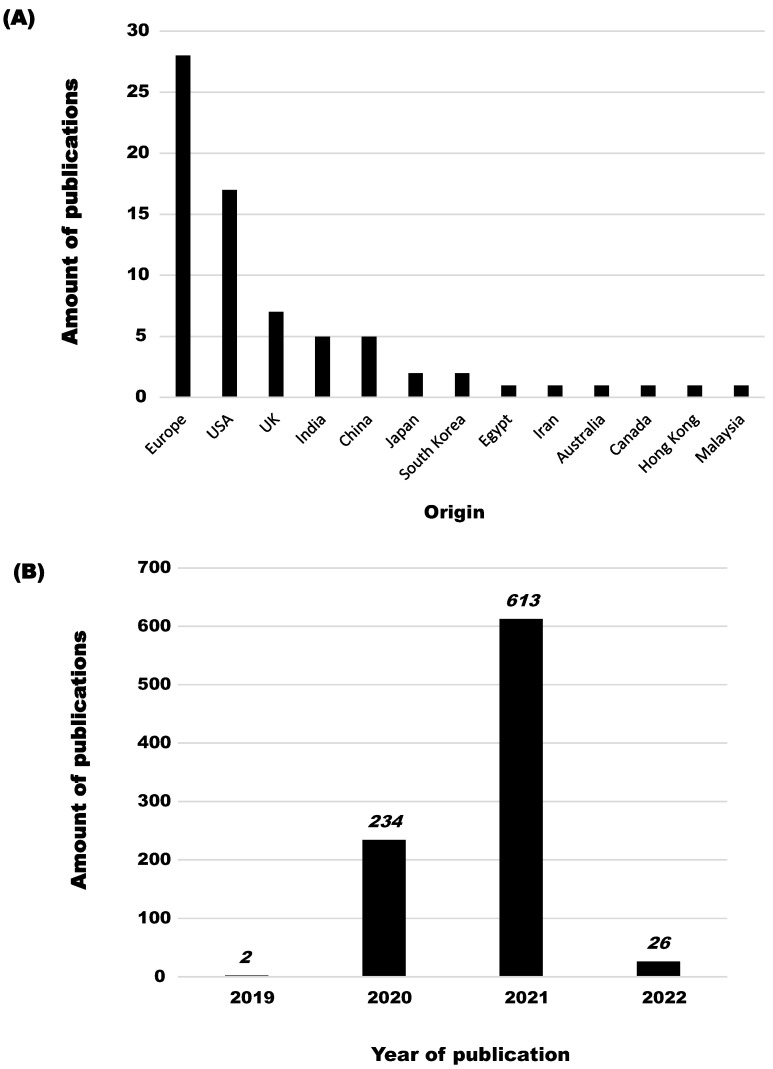
Origin of publications included in this systematic review (**A**), and publication activity by year of records screened (**B**).

First, 1310 records were retrieved from the aforementioned databases. After elimination of duplicate records (419), 891 were screened based on their title. The remaining records were checked for availability, eliminating a total of 102 publications, which were either unavailable, in pre-print, or not in English. The remaining 269 records were screened by abstracts, and by the first and the two last authors, excluding 198 records based on the selection criteria described above. When there was doubt, a consensus meeting between the three protagonists was held to decide on final exclusion. In the end, 69 articles and reviews were kept and included in this work (Appendix B). In order to conceptualize the overwhelming amount of publications surrounding this thematic, Figure 2 illustrates the publication activity surrounding the pandemic and, more specifically, the history linked to the search strings described above, and the country of origin of the selected publications. While most of the output concerning indoor transmission originates from Europe and the USA, a global effort, as reflected by available publications from around the world, must be acknowledged. This confirms that the COVID-19 pandemic is indeed a global pandemic, and that only by striving to eliminate the SARS-CoV-2 virus globally can an end be found to this worldwide problem.

### 3.2. Description of Aerosol and Droplet Transmission

To understand aerosol and droplet transmission, it is important to grasp the mechanism behind the production of infectious droplets. Infectious droplets of varying sizes, loaded with salts and viral particles, are produced by infected individuals when performing respiratory activities. Coronaviruses are enveloped viruses which survive in the aqueous phase of such respiratory droplets [23]. The survival time of the virus is dependent on the lifetime of the droplets; the dynamics of these droplets will depend on their radius. The larger the droplet radius, the smaller the suspension time of a single droplet [24,25]. The settling times of these particles in still air can be predicted accurately using existing physical models, such as Stokes’ law [9,24,25,26]. However, most enclosed spaces present ambient air currents, and these currents are exacerbated by the presence of natural or mechanical ventilation systems. Droplets with a radius between 50–100 µm have a high probability of falling within 1–2 m from the infected emitter. During more intense respiratory activities by the emitter, such as coughing, sneezing or loud talking, these droplets can be carried beyond 2 m [16]. Smaller droplets between 5–10 µm have a much lower settling speed, and take around 8–10 min to fall from a height of 1.5 m [27]. These droplets can stay suspended in the air for much longer when air currents are active. For even smaller particles of under 5 µm in radius, air flows are the main carriers, and these droplets can stay suspended for a very long time [8,9,23]. In addition, in cold and humid conditions, larger particles up to 10 µm have shown to be able to travel longer and further in the air [27].

The size distribution of the produced particles varies, and depends mostly on the activity of the emitter. For respiratory activities, such as breathing, talking, and coughing, the majority of aerosols have a diameter of less than 5 µm, and a large fraction has a diameter of under 1 µm [8]. Bazant et al. [16] analyzed droplets emitted in the course of various activities (such as breathing, whispering, speaking, and singing), and showed that different respiratory activities produce differently sized particles. They concluded that, for example, nose breathing produces less and smaller droplets than breathing from the mouth, and that singing loudly produces a significant number of larger droplets.

Fine infectious aerosols can travel for long distances in the air and can carry a sufficiently large viral load to cause infection in healthy subjects [23,28]. These infectious aerosols can originate from fine aerosols carrying sufficient viral loads [29], or be produced by larger particles with larger viral loads, who have undergone partial evaporation before settling to the ground [28].

Thus, it is crucial to understand both the evaporation mechanisms and the parameters influencing them. Evaporation time depends strongly on the temperature and humidity of the ambient air, impacting the behavior of respiratory droplets [23,27,28,30,31]. The influences of these factors will be discussed below. Table 2 summarizes the different parameters used in the models described in this work.

### 3.3. The Wells Riley Model and Its Successive Improvements

Various mathematical models have been used to describe the spread of viral infections. Among these, the Wells–Riley model has been widely used to determine the probability of infection *P*, following a Poisson law (in Equation (1)), and is well-accepted for the description of the airborne spread of viral particles [32].

(1) $$P=1-exp\left(\frac{-N_{i}\times Q\times q\times t}{V\times \lambda_{a}}\right)=\frac{Number\;of\;new\;cases}{Number\;of\;susceptibles}$$

where *Ni* represents the number of infectors, *V* the volume of a room, and *q*, *Q*, *t*, and *λ_a_*, the rate of production of infectious quanta per unit of time per infector, the pulmonary ventilation rate, the time of exposure, and the rate of room ventilation, respectively. The term *N_i_* × *q* × *p* × *t*/*Q* is equivalent to the dose of exposure. It is interesting to note that there is no distance-related variable in this model. Indeed, assuming a closed a well-mixed place, a healthy individual is no safer from infection from 10 m than from 1 m away from an infected individual. The assumption is made that any infectious particle emitted from an infected individual has an equal chance of being anywhere in the room at any given time. The original Wells–Riley model relies on other assumptions, which underlines some of its limitations. In this model, transmission is considered to be exclusively airborne, thus ignoring transmission through fomites. Moreover, particle loss rate is based solely on ventilation, ignoring decay through air filtration, viral deactivation, and particle sedimentation.

Shen and colleagues elaborated a more complete model for the estimation of this probability, based on the original Wells–Riley model [33]. In this model, a number of parameters allowing a more precise estimation of the airborne-infection risk are included (Equation (2)):

(2) $$P=1-exp\left(-R_{s}R_{I}\frac{N_{i}\times q\times p\times t}{V\times \lambda }\right)$$

where, additional terms *R_S_* and *R_I_* represent the fraction of infectious particles penetrating through the masks of the susceptible and infected population, respectively. These depend on the mask-filtration efficiency *η* and the fraction of time the mask is used over the exposure period *t* (Equation (3)):

(3) R=1−η×t 

The volume of the room is represented by *V*, and *λ* represents the particle loss rate, which is composed of multiple factors (Equation (4)):

(4) R=1−η×t 

The amount of fresh air present in the room not only depends on a ventilation rate *λ_a_*, but also on the renewal of the air already present in the room, dictated by *λ_f_*, *λ_v_*, and *λ_s_*, respectively, the air filtration rate, viral deactivation rate, and particle sedimentation rate. The introduction of these different terms allows the model to account for more removal processes other than ventilation rate, which is the only process described in the Wells–Riley model. For example, an additional ventilation factor can be introduced to account for the differences of airflow within a room. In the same manner, a filtration-efficiency factor can be added in function of the air filters present, and pathogen removal factors can be added when using germicidal technologies. Finally, the sedimentation rate depends on the size distribution of the particles present.

### 3.4. Quantum of Infection and Quantum Generation Rate

The Wells–Riley model also introduces the term quantum of infection. A quantum of infection is defined as the number of infectious droplet nuclei or the infectious dose required to infect 1 − 1/e, i.e., 63.2% of susceptible persons in an enclosed space [32,34]. Since the beginning of the pandemic, emergence of new SARS-CoV-2 variants has been documented. Some of these variants are more contagious than others, which means that one quantum of infection for the more contagious variants will contain a lesser infectious dose. Burridge et al. discuss the fact that the B1.1.7 variant (also known as the Alpha variant) could be 70% more infectious than the “pre-existing” strains; this suggests an according increase in the quantum generation rate of 70% [12].

At this point, it is important consider the quantum generation rate or quantum emission rate (*q*) and the concentration of infectious quanta in the exhaled air (*Cq*) associated with it. A related term is the pulmonary ventilation rate *Q*, which allows linkage of both terms through the following formula: *q* = *Q* × *Cq*. These terms represent the number of infectious particles that an infected individual will produce. Buonnano et al. showed that high quantum generation rates (>100 h^−1^) can be reached by asymptomatic carriers performing certain activities, whereas a symptomatic carrier in resting conditions can achieve quantum generation rates as low as 1 h^−1^ [34]. Dai and Zhao [35] report rates between 14 h^−1^ and 48 h^−1^. It is plain to see that these values can vary significantly. Indeed, the number of quanta produced depends heavily on the type of activity a given subject is performing and is also highly variable depending on the stage of the disease. A high number of airborne particles is produced when an individual speaks loudly or sings. The Skagit Valley Chorale superspreading event is an example of high exposure to the virus due to high quantum generating activities [9]. Studies investigating this event estimate generation rates of up to 970 h^−1^ due to loud singing of infected individuals [36]. Bazant et al. [16] analyzed various expiratory activities ranging from breathing to speaking to singing, and showed that the associated quantum emission rates vary significantly. The monitoring and control of activities performed indoors thus seems crucial to avoid high infection risks. Moreover, this parameter does not only vary depending on the activity of the infected subject, but also in the function of the infectivity of the virus, as mentioned above. As the quantum emission rate varies depending on the activity performed by the infector, it will not be a constant over time. While use of a constant emission rate can simplify the models used, Kurnitski et al. propose a method for the calculation of an average emission rate over time, allowing for a more precise estimation of the infection risks (Equation (5)) [37].

(5) $$\bar{C_{q}}=\frac{q}{\lambda V}\left[1-\frac{1}{\lambda t}\left(1-e^{-\lambda t}\right)\right]$$

where ($\bar{C_{q}}$) is the time-average concentration of infectious quanta in the air, *q* is the quanta emission rate, *λ* is the particle loss rate, *V* is the volume of the room, and *t* the time.

This model of Kurnitski et al. relies a full mixing assumption, meaning that inside a well-mixed room, respiratory aerosol gets distributed in a homogeneous way. This can create certain inaccuracies because viral concentration is not necessarily equal in the whole room when considering large volumes and/or large floor areas.

### 3.5. Risk Factor Assessment

Another model, based on the Wells–Riley model, was developed by Bazant et al. [16], and defines a risk tolerance ε in function of the cumulative exposure time *t* (Equation (6)).

(6) $$\varepsilon =\frac{N\times t\times Q^{2}\times R_{S}\times R_{I}\times C_{q}\times s_{r}}{\lambda \times V}$$

where, *Q* represents the pulmonary ventilation rate, *N* is the number of susceptible individuals present in the room, *R_S_* and *R_I_* represent the fraction of infectious particles going through the masks of susceptible individuals and infectors, respectively, *Cq* is the concentration of infectious quanta in the exhaled air, *s_r_* is a transmissibility factor, *λ* is the particle loss rate, and *V* the volume of the room. This risk tolerance is chosen to bound the probability of one infection [26]. Again, the model of Bazant et al. relies solely on airborne transmission, neglecting transmission through fomites. It also does not account for room occupants’ arrangement and relies on the assumption of a well-mixed room.

### 3.6. Influence of Temperature and Humidity on Airborne Spread

The transmission modes for respiratory viruses by droplets of varying sizes have already been described above. The size distribution of droplets is not constant, but varies according to certain parameters, including air temperature and humidity [38]. Moreover, the control of the temperature and humidity of ambient air in indoor settings is crucial for various reasons. First, comfortable conditions should be established for places where people spend a considerable amount of time. Second, the humidity should be controlled to avoid the proliferation of mold and moisture. The relative humidity (RH) should, therefore, be kept below 80% [8,31]. Third, various studies show that the human mucous membranes become more vulnerable at low relative RH values (below 30%) [6,8,20,31]. Finally, temperature and humidity influence the inactivation rate of viruses [8,11,39]. We can describe the decay of a virus following a simple formula (Equation (7)):

(7) $$C\left(t\right)=C_{0}\times e^{-\lambda_{v}t}\;$$

where *C* is the virus concentration at time *t*, and *C*_0_ the initial virus concentration. Previous studies have shown that the viral inactivation rate *λ_v_* depends on the RH. Indeed, various reports show that this deactivation rate tends to be very high at intermediate values of RH, when the virus is most exposed to salts and solutes [6,16]. These conditions occur at RH values between 40% and 60%. The RH of indoor spaces should thus be kept around these values to limit the airborne survival time of the virus [40]. Temperature regulation also performs an important role, as it has been shown that increasing temperatures significantly reduce virus half-life [40,41,42], while low temperatures allow a prolonged virus survival time [27].

When aerosols are emitted, droplets of different radii are emitted. If the radius is smaller, the droplets will remain suspended in the air for a longer time. As mentioned above, temperature and humidity have an influence on the evaporation of droplets, thus impacting the size of droplets suspended in the air. More specifically, hot and dry conditions will accelerate the evaporation of droplets [43]. When large droplets evaporate and shrink, the concentration of viral particles within the same droplet increases. This process leads to the existence of small airborne particles with high viral loads, potentially able to infect healthy subjects at larger distances, as described above.

### 3.7. CO_2_ as an Indicator of the Room Ventilation

Indoor carbon dioxide (CO_2_) monitoring is crucial for the comfort of the occupants. Indeed, too high CO_2_ levels can lead to loss of concentration and even adverse health effects [44]. Additionally, the CO_2_-levels of a room can be used as an indicator of the ventilation of said room and as previously stated, a good ventilation of rooms is crucial for the mitigation of indoor airborne spread of viruses. Moreover, CO_2_ is a marker of exhaled air, and can thus be incorporated into infection probability calculations through the Wells–Riley model [26,45]. When an infected individual enters a room, infected particles accumulate, contributing to a higher infection risk for other occupants. Thus, ventilation is key, not only to lower the risk of infection, but also to lower exposure to air pollutants that can cause other diseases.

Indoor air quality guidelines dictate that CO_2_ levels above 2000 ppm could be potentially dangerous for the occupants, and recommend concentrations below 1000 ppm [44,46,47,48]. Calculations of excess CO_2_ in a room can be made through the following formula, where the excess of CO_2_ represents the difference between the indoor and outdoor CO_2_ concentrations [26]:

(8) $$\Delta \left(CO_{2}\right)=\frac{N\times Q\times k}{\lambda_{a}\times V}\;$$

where, *k* represents the concentration of CO_2_ in the exhaled air, on average around 38,000 ppm (3.8%). Equation (8) can be inserted into Equation (6), in order to assess to excess CO_2_ in function of a certain risk tolerance:

(9) $$\Delta <\frac{\varepsilon \times k}{t\times Q\times R_{S}\times R_{I}\times C_{q}}$$

This equation is especially interesting because it does not depend on the volume of the room or on the number of occupants. Moreover, knowing the ventilation rates is not required here as the calculated CO_2_-levels serve as proxy for these rates. Using Equation (9), we can establish a list of typical scenarios, and assess the maximum CO_2_ levels needed to stay below a certain risk of infection using 400 ppm as a base value for outside CO_2_ concentration. These maximum allowed CO_2_-levels are listed in Table 3, using a risk factor ε of 10%. Values for *Q* for adult individuals have been reported by Shen et al. [38] as follows: 0.3 for sedentary activities, 1.6 for moderate-intensity activities, and 3.0 for high-intensity activities. It is important to note that the results obtained through the aforementioned equations rely on certain assumptions and simplifications [20]. It is assumed that the air in the room is well-mixed. Mask wearing is assumed to be uniform across susceptible individuals, and constant over time. Values for *Q* and *Cq* are averages and sourced from various publications. There is no other source of infectious quanta apart from the considered infector. Resuspension of sedimented particles is neglected. Moreover, for these models, the studied spaces are assumed to be in a steady state. Kurnitski et al. developed a more complex model and showed that assuming a steady state only leads to a small underestimation of the infection probabilities [37].

Based on the different scenarios shown in Table 3, it is clear that the activity of the infected individual performs a crucial role regarding the risk of infection and, indeed, regarding the ventilation level required to mitigate this risk. The high values shown in Table 3 should not be regarded as target values, but only serve to show that infection risk is very low in these specific scenarios. The risk of infection tends to be very low when the infector engages in non-intensive activities, wears a mask, and breathes lightly. This shows why classrooms and other meeting spaces are relatively safe settings when masks are worn. However, when the infector performs a vocalizing activity, such as teaching, speaking loudly, or is performing physical activities, with no mask, the risk of infection increases significantly, and ventilation may not suffice to effectively mitigate this risk. It should be remembered that these conclusions are based on a situation where the air inside the rooms is well-mixed. Additionally, while CO_2_ can be an adequate proxy for suspended pollutants, it does not always adopt the same dynamics as infectious droplets. The risk assessment described here can be a good way to quickly estimate if an environment is safe or not, but should not be adopted on its own for a more fine-tuned mitigation strategy. For this, CO_2_-monitoring with adequately placed CO_2_ meters should be coupled with the analysis of the airflow dynamics, ventilation, filtration, and recirculation systems.

### 3.8. Heating, Ventilation and Air Conditioning (Indoor Air Quality Control Systems)

Based on what has already been stated, it is clear that the monitoring of the indoor air quality (IAQ) is crucial in order to prevent indoor transmission of the SARS-CoV-2 virus or any other airborne pathogen [42]. The implementation of IAQ control systems can act on different parameters of the models described above. Ventilation rate *λ_a_*, sedimentation rate *λ_s_*, filtration rate *λ_f_*, and viral deactivation rate *λ_v_* are all factors strongly influencing the transmission probability of SARS-CoV-2, and can be controlled through IAQ control systems.

Displacement ventilation, i.e., when outdoor air is supplied from floor-level diffusers and extracted at ceiling height, could be the most efficient strategy for reducing transmission probabilities [47]. Indeed, rising airflows allow the removal of infectious suspended particles from the breathing zones of the occupants and remove warm contaminated air near the ceiling. Air recirculation should be avoided as this reintroduces contaminated air into a room instead of bringing in fresh and clean air, unless equipped with efficient air filters [49]. The implementation of physical barriers, such as plexiglass windows designed to block the spray of larger droplets, can have adverse effects on ventilation as they prevent airflow, trap infectious suspended particles in the breathing zone, and can thus increase risk of infection [8]. To maintain a good ventilation, as monitored by CO_2_ meters, it is important to respect a certain ventilation rate. A minimum of six air changes per hour (ACH) is recommended to maintain a satisfying indoor air quality, and to lower the risk of infection [7,42,48,50]. However, in small rooms, even when respecting required ACH, the proximity of the occupants can still lead to high risk of infection [51]. It is not always easy to determine the exact ACH of a room, especially when multiple ventilation systems are in use. However, the ACH can be calculated through the monitoring of CO_2_ levels. Indeed, Aguilar et al. reported a method for determining the ACH based on CO_2_ decay curves [52]. The following formula was proposed:

(10) $$ACH=\frac{-\ln\frac{C_{end}-C_{outdoor}}{C_{start}-C_{outdoor}}}{t_{end}-t_{start}}$$

where *C_end_* is the CO_2_ concentration at the end of the decay curve, *C_outdoor_* is the outdoor CO_2_ concentration, *C_start_* the concentration at the start of the decay curve, tend is the end time of the decay curve, and *t_start_* is the start time of the decay curve.

The recommended value of six ACH can be hard to achieve, but the greatest possible air change will be beneficial. Overall, ACH is not the most reliable metric for ensuring good air quality. Filters can be introduced to clean the outflowing air, and remove particles and infectious bioaerosols. High Efficiency Particulate Air (HEPA) filters remove up to 99.9% of aerosol particles [16]. Portable Air Cleaners (PAC) equipped with HEPA filters can be an efficient and easy to deploy way to control IAQ, and to mitigate risk of infection [42,49]. In order to achieve a satisfying efficiency in the fight against infectious agents, these should be able to remove particles in the range of 0.1 µm to 1 µm of diameter [53]. Short-wave ultraviolet (UV-C) irradiation filters, photocatalytic filters, and ozone inactivation technologies have also showed promising results when integrated into IAQ control systems [53,54,55].

Spena et al. recently reported various viral load survival rates (VLSR) in function of the specific enthalpy of the ambient (moist) air [31]. This study showed that between 50 kJ/kg and 60 kJ/kg the VLSR is kept to a minimum. Moreover, for optimal comfort conditions in domestic and office-like spaces, the American Society of Heating, Refrigerating, and Air-conditioning Engineers (ASHRAE) has a recommended zone in the psychrometric chart of moist air [56]. An ideal zone in terms of relative humidity, temperature, and specific enthalpy for the comfort of the occupants on the one hand, and a minimal exposure risk of infection on the other hand is determined as: a RH between 40–80%, and a temperature between 20–25 °C. These conditions can easily be obtained through appropriate heating, ventilation, and air-conditioning (HVAC) appliances, which monitor and control the Indoor Air Quality (IAQ).

### 3.9. Natural Ventilation and Manual Operation of Doors and Windows in Enclosed Spaces

Many buildings and indoor spaces are not equipped with ventilation systems or other air-conditioning appliances, and rely solely on the manual opening and closing of doors and windows for good ventilation of these spaces. This is usually the case in old office buildings or schools, which have neither the financial nor structural possibilities of adopting automated ventilation strategies. Naturally, keeping the CO_2_ level, and thus the risk of infection, at a minimum, becomes a challenge under these conditions, and in the presence of infectious occupants, these spaces become environments with high exposure to airborne viral particles [13]. Several studies have investigated the air quality fluctuation in classrooms depending on the room occupation, the duration of classes or other activities, and the natural ventilation possibilities. These reports notably show that spontaneous door and window ventilation (meaning the spontaneous opening of doors and windows by the occupants) in countries with cold winters can lead to unacceptably high CO_2_ levels. The implementation of shorter room occupation time and fixed breaks, with students leaving the classroom, during which natural ventilation is made possible by opening all doors and windows, proved to be effective in keeping the CO_2_ level low enough [17,44,47,57]. Most of these studies, describe between 5–30 min to be needed to return to base CO_2_ values, depending on the number of doors and windows opened [58]. Ventilation via doors and windows during the occupation of rooms should be adopted with caution, since this can provoke uncontrolled airflows that may guide potentially infectious particles towards the breathing zones of the room occupants [59].

### 3.10. Ultra Violet Radiation, Photocatalytic Filters and Other Germicidal Compounds

Several studies discuss the use of UV-C radiation (200–280 nm) in order to inactivate viral particles present in the air; UV-C has already shown its effectiveness in the fight against measles, tuberculosis, and several other airborne viruses [54,55]. While UV radiation can prove harmful to human skin and must not be deployed when a room is occupied [55,56]), irradiation during periods of vacancy is a viable strategy. The periodic illumination of intermittently occupied spaces (or public transport vehicles, such as busses or trains) can prove beneficial in reducing viral loads of indoor air [55]. When using UV radiation against viruses and other pathogens, it is crucial to administer lethal doses since unsuccessfully or insufficiently exposed pathogens may develop resistances against subsequent radiation. Ideally, UV-light should be used together with photocatalysts in the context of an air filtration technology referred to as UV photocatalytic oxidation (PCO). In such systems, UV radiation allows for a direct inactivation of genetic material and proteins inside viruses and bacteria, while UV-activated photocatalytic surfaces produce virucidal oxidative radicals, thus providing an effective synergy [55]. Such filters can prove an efficient, cheap, easily deployable, and scalable technology to act upon airborne viral spread. They can be deployed inside ventilation ducts, efficiently deactivating viral particles inside; they can also be used to inactivate pathogens on frequently touched objects and surfaces, such as keypads, door handles, and handrails.

### 3.11. Mitigation Measures

**Figure 3:**
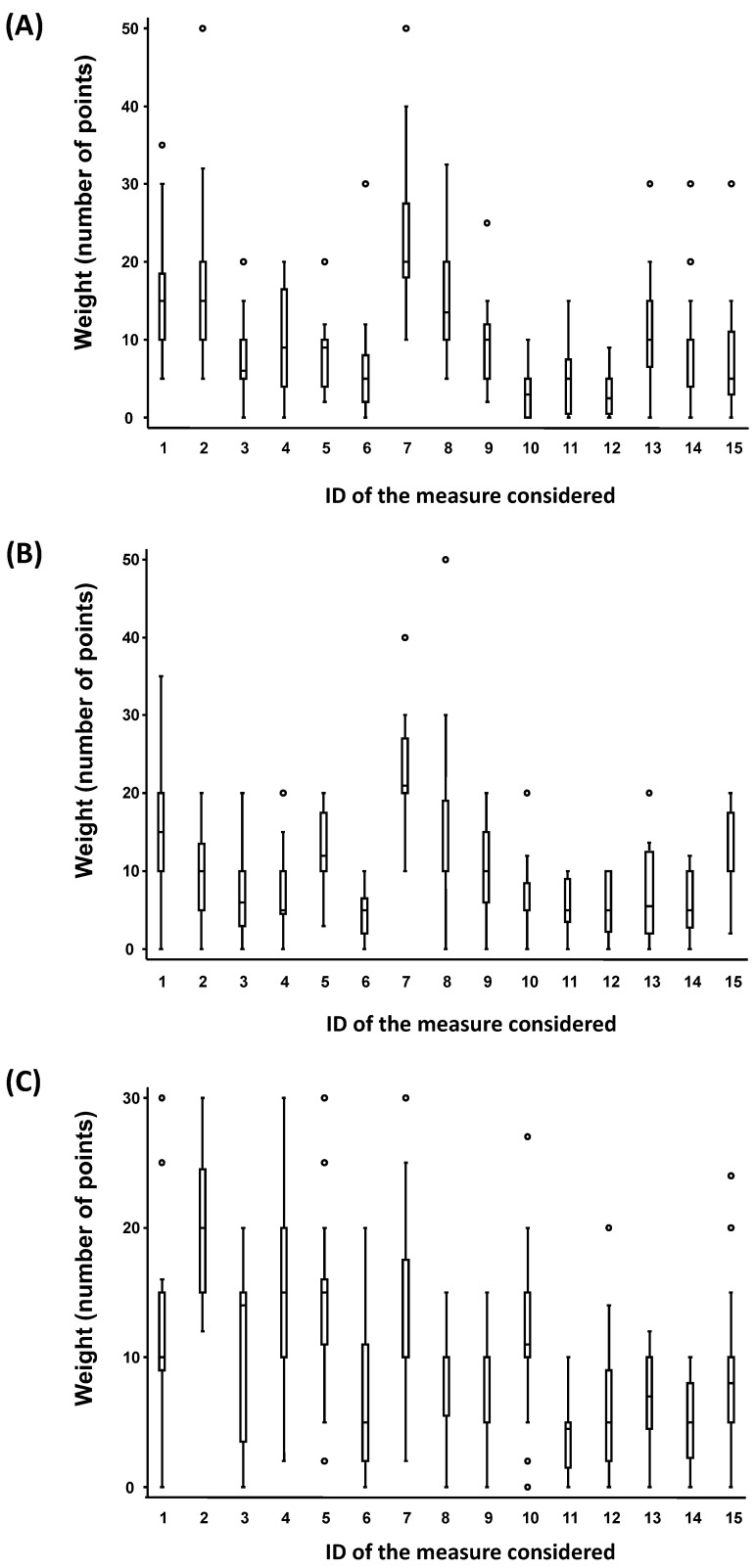
(**A**) Efficacy, (**B**) feasibility, and (**C**) acceptability of the considered measures. Legend: The line inside each rectangle represents the median of the score distribution between the different experts; the solid lines below and above each rectangle represent, respectively, the first and the third quartiles; adjacent lines to the whiskers represent the limits of the 95% confidence interval; small circles represent outside values. ID of the measure considered are numbers of mitigation measures that were presented in the second column of Table 4.

Table 4 shows 15 measures that can be implemented to mitigate the risk of infection in enclosed spaces, many of which can act on a variety of parameters influencing airborne transmission. In order to assess the impact of these different measures, a panel of multi-disciplinary experts (*N* = 20 co-authors of this paper) was asked to judge the efficiency, feasibility, and acceptability of the proposed measures. The expects were asked to distribute 150 points among the 15 measures. The weight distribution of these measures for the efficacy, feasibility, and acceptability is shown in Figure 3. To test the robustness of the expert elicitation, sensitivity analyses were performed through the jackknife resampling technique, and showed no significant variation in the rankings of the measures when omitting one expert.

According to this panel of experts, the most efficient mitigation strategies rely on accentuated ventilation, both mechanical or natural, the use of face masks, and the reduction in the number of occupants in a room. These measures also rank highly both in feasibility and acceptability. Air quality monitoring (through, e.g., CO_2_ meters, TVOC meters, and PM detectors) is also considered fairly efficient, acceptable, and feasible. Among the measures taken prior to room occupancy, only the refusal of symptomatic individuals stands out. Other measures are judged either not efficient, not acceptable, not feasible, or a combination of all three. Even though some governments (e.g., France, Belgium, and Italy) require a COVID-19 certificate for access to many public spaces (e.g., events, restaurants, and hotels), this strategy is not considered very efficient, and causes increasing disgruntlement among certain parts of the populations. The COVID-19 certificates serve as proof of vaccination or, in some cases, proof of recent recovery from COVDI-19 infection. Surprisingly, solely refusing entry to unvaccinated individuals is not generally considered an important measure compared to others. This might be due to the fact that, up until now, vaccination strategies have not provided the effect expected on the fight against the pandemic. Third doses are being administrated in multiple countries, but nations keep struggling with contagion numbers that will not stay low. Additionally, temperature and relative humidity control are subjectively not considered effective tools for mitigating the infection risks indoors, even though this systematic review describes it to be a good measure to control, and increase inactivation of viral particles present inside.

Previously described HVAC appliances are not only useful for the ventilation of rooms, but can also to help monitor room temperature and humidity. By doing so, it is possible to achieve optimal conditions not only for viral inactivation, but also for human comfort. Indeed, Equation (2) shows that airborne transmission is in part dictated by the particle loss rate *λ*. By introducing a higher viral deactivation rate, *λ_v_*, *λ* will in turn increase, and by consequence, the probability of infection *P* will decrease. In addition, the control of temperature and humidity also allows to have a certain grip on the droplet evaporation dynamics, which in turn influence the concentration of infectious particles suspended in the air. Germicidal appliances, such as UV-C radiation, catalytic filters, and ozone, can also help increase the viral inactivation rate and decrease the concentration of viral particles present in the air. While external UV-C lighting may be considered neither efficient, feasible nor acceptable, equipping air filters internally with UV-C technology might be a less intrusive and more effective way for deactivating viral airborne particles.

Finally, the implementation of HVAC appliances provides effective ways of fighting infection risks through ventilation. However, it is important to investigate air flow dynamics in rooms equipped, or to be equipped, with ventilation systems. Indeed, badly placed vents can lead to stratification of air layers, which may cause an increased risk for some individuals in the room [60]. CO_2_ meters allow for an effective monitoring of the ventilation of indoor spaces and could be used to send feedback to HVAC appliances. The necessary ACH can be calculated (through Equation (10)), and be applied to keep below a certain infection risk. It is important to note that the installation of HVAC appliances or large-scale modification of pre-existing equipment entails vast financial costs and potentially long implementation times. For older buildings, such systems can prove impossible to install [61]. One solution to bypass the structural impossibility of installing HVAC systems is to utilize portable air cleaners. These can be transported, installed easily inside rooms, and provide an effective way of removing bioaerosols in air [53,62]. Moreover, the versatility of these appliances makes it possible to direct airflows by strategical positioning, and, thus, to avoid the redirection of infectious particles into the breathing zone of room occupants [42]. Air purifiers should be carefully selected and must provide sufficient airflow with a minimal noise level.

Wherever technological solutions are not available, human behavior must be adapted to mitigate airborne spread, and social measures have to be taken. For example, an effective measure can be to halve the usual occupancy of indoor spaces [49]. Equation (2) shows the probability of infection for a person entering the room. When considering a group of people, we can multiply this equation by the amount of susceptible *N*, in order to obtain the probability that at least one infection occurs. This probability is divided by a factor n when the amount of people in the room is divided by a factor n. Moreover, dividing the number of occupants also reduces the chance of having infectors present. Thus, if we halve the number of participants in a classroom, for example, the probability that at least one infection occurs will be divided by a factor of 4. Mechanical ventilation rules can be put in place and are already applied in various schools around Europe. By manually opening doors and windows and emptying classrooms during breaks, as previously discussed, CO_2_ level can be kept below a certain level, thus efficiently mitigating the risk of infection during classes [44,46,50,58,63,64].

In sum, infection control strategies through ventilation of rooms strongly depend on the characteristics of the room, and on the use of said room. In order to implement efficient mitigation strategies, an analysis of each separate room should be performed, and the most feasible and efficient ventilation system should be adopted. Various factors, such as available vents, room height, room volume, room use, economical and energetical requirements, and structural characteristics, should be considered in these analyses.

Monitoring the activities of people present in enclosed spaces can also be key to lowering the risk of infection. Loud speaking, singing, screaming, or other intense vocalizing activities will produce more respiratory droplets, and thus, increase the infectious quanta concentration *Cq* in the air. Asking speakers indoors to keep their voice down and using devices, such as microphones, will reduce the risk of infection. Additionally, avoiding physical exercise in indoor spaces or lowering respiration rates before entering enclosed environments can also prove effective. The reduction in the time spent inside may also be added to these measures. However, as Table 4 shows, the occupants of these spaces may be reluctant to drastically change the way they operate inside.

Another means for controlling the number of infectors initially present in the room is an increased screening of the participants. Indeed, controlling vaccination passes or other COVID-19 certificates, or refusing the access to certain spaces for people presenting symptoms (e.g., body temperature) will undoubtedly reduce the number of potential infectors present in the room, thus effectively reducing the risk of transmission. However, such measures will find great resistance from certain subsets of populations as they have a large impact on personal freedoms of affected individuals.

Finally, wearing a mask has a large impact on the infection risk [65]. Indeed, when both infectors and susceptible wear masks (represented by *R_i_* and *R_s_* in the aforementioned models), the infection risk is greatly reduced, and could potentially be brought down to 0 if the fraction of aerosols filtered by the masks tends to 100%. A good compliance with mask wearing rules (covering both mouth and nose) should not be underestimated, and, if possible, subjects should keep their masks on at all times to mitigate the risk of infection. Single-use masks break down rapidly and their filtration efficiency plummets after multiple uses. The use of higher-performance face masks (N95), face masks equipped with virucidal properties [66,67], or the decontamination of used masks [68], could prove simple and cost-effective ways of reducing the risk of infection. Thus, the use of face-masks should not be underestimated, and authorities should encourage people to wear face masks at all times.

As a final note, it is worth mentioning that these mitigation measures and other recommendations can also be applied to the control of any other infectious bioaerosol.

### 3.12. Seasonality of the SARS-CoV-2 Virus

Above findings clearly hint at a seasonal character of the SARS-CoV-2 virus. Indeed, seasonal variations bring about changes in humidity and temperature, but also impact sunlight intensity, host immune responses, and human behavior. These changes in human, environmental, and viral factors induce changes in the evolution of the current pandemic [6]. Indeed, it is unsurprising that infection and mortality numbers tend to be lower during summer periods [2], when higher temperatures, low RH, and abundant sunlight enhance the viral inactivation, and ventilation measures are well respected in order to keep indoor environments cool and breathable. Inversely, infection numbers peak during colder periods, when lower temperatures and higher RH favor the viral load survival rate, and people neglect proper ventilation procedures in order to keep warm [44,64]. This trend is also recognized in other respiratory viruses, such as SARS-CoV-1, influenza, and human respiratory syncytial virus (RSV), which show peak incidence rates during winter months [6,8]. The original 2019/2020 SARS-CoV-2 outbreak during winter months (December–January) is hence unsurprising. Moriyama et al. recently presented a list of recommendations to limit viral spread during winter months. These include the humidification of indoor air and the supplementation of vitamin D to compensate for decreasing daylight [6].

As suggested in Table 4, seasonal changes will have an impact on the efficiency, feasibility or acceptability of some measures. An inquiry performed at the University of Liège asked the occupants of classrooms, university restaurants, libraries, and other indoor spaces to report on the opening of doors and windows during a period when students and personnel were allowed to return to the university (September 2021 to January 2022). The participants were asked if, yes or no, the doors and windows were opened during at least 50% of the occupation time. Appendix C plots the opening of the doors and windows together with the temperatures recorded during this period. This data shows that when temperatures drop, occupants are more reluctant to open windows and prefer opening doors as this brings less thermal discomfort. Additionally, temperature and humidity control of indoor spaces has to be operated differently depending on the seasonal variations. Indeed, outside conditions have an impact on inside temperature and RH. Finally, colder months increase symptoms of respiratory infections among occupants. These symptoms are similar to the ones presented by SARS-CoV-2 infected individuals. Thus, refusing access to certain spaces for individuals presenting COVID-19-related symptoms may be an efficient measure, but will exclude non-contaminated subjects (false positive).

Seasonal variations also occasion a variation in transmission modes. While direct droplet transmission and fomite transmission are more likely during the summer, airborne transmission in closed spaces is the main mode of transmission during the winter [6]. Studies by Kwon et al. recently demonstrated an elevated stability of the SARS-CoV-2 virus on surfaces and biological fluids during northern hemisphere winter seasons, and showed extended stability of the virus to be one of its key characteristics [69]. Their data showed the virus to remain infectious for 2 days in nasal mucus and sputum during summer conditions, whereas can remain stable for up to 21 days in winter conditions.

## 4. Conclusions and Perspectives

The different transmission pathways of SARS-CoV-2 have been documented since the beginning of the pandemic. While airborne transmission through suspended bioaerosols was initially widely debated, today, a consensus exists within the scientific community is that this pathway should not be neglected, especially in indoor environments. This review documents the mechanism of this transmission mode, and the parameters affecting it. Airborne transmission occurs when respiratory droplets are exhaled by an infector. Since droplet lifetime and behavior are subject to environmental conditions, the airborne transmission mode is strongly dependent on indoor temperature and humidity. Moreover, environmental conditions also perform an important role in the survival time of the virus in the air and on surfaces. Thus, an efficient and strategic control of these conditions is crucial in order to reduce the risk of infection. Indoor relative humidity should stay in the range of 40% to 80%, and temperature should be kept above 20 °C, in order to minimize risk of infection, while maintaining comfortable conditions for the occupants of considered rooms.

Of the various mathematical models designed to describe droplet transmission and risk of infection, the Wells–Riley model seems to be the most accurate. Here, we document this model, its different parameters, and its successive improvements. An important parameter included in the model is linked to the emission rate of infectious quanta, i.e., the amount of infectious viral particles emitted by infected individuals. This particular parameter strongly depends on the activity of the infector but is also dependent on the variant of the virus. Different variants of the virus can present different quantum emission rates. Therefore, the possibility that certain more contagious variants can be transmitted through hitherto neglected infection routes should not be excluded. Thorough investigations of infectivity and quantum emission rates for different variants of the SARS-CoV-2 virus should be performed, and different measures may be proposed and adapted depending on the prevalence of different variants.

Ventilation is a key factor for the risk assessment through the mathematical models described here. As has shown via various scenarios, using CO_2_ levels as an indicator of the ventilation of a room, and thus of the risk infection in real time, can be an easy and effective way for monitoring the risk related to certain activities performed indoors. Strategically installing CO_2_ meters can allow the occupants of a room to monitor current CO_2_ levels, and to adapt ventilation protocols if CO_2_ levels rise to unwanted levels. Ventilation can be performed using mechanical HVAC appliances, which allow a precise control of the air changes in a room. To keep infection risks low, a minimum of six air changes per hour is recommended, and air recirculation should be avoided. Furthermore, HVAC appliances allow the control of the temperature and humidity of rooms, and can be equipped with filters to remove infectious particles from the air. In the absence of mechanical ventilation, manual ventilation, by opening doors and windows, must occur. Promising technologies have lately emerged in the form of SARS-CoV-2 detection devices, which can detect viral particles in the air and even distinguish SARS-CoV-2 variants. These portable devices can either be used by individuals to analyze exhaled air, or can be placed inside larger spaces to detect aerosolized viral particles. While such technologies are relatively new (their efficiency and potential for large-scale deployment remain to be demonstrated), they may prove crucial for allowing risk-free indoor activities.

A panel of 20 multi-disciplinary experts ranked 15 proposed mitigation measures according to their estimated efficiency, feasibility, and acceptability. A summary of the most and least efficient, feasible and acceptable measures is shown in Table 5. The measures that stand out in all categories are ventilation of rooms, mask wearing, and air quality monitoring. Infection control strategies in indoor environments should place a strong accent on these measures and their optimal combination.

Finally, we have shown that the “behavior” of SARS-CoV-2 strongly depends on seasonal conditions (among which temperature and relative humidity). Since winter months present a higher risk of infection due to lower viral inactivation and increased time spent indoors, infection control-strategies should be adapted in function of the season.

## Figures and Tables

**Table 1:** Definitions related to the airborne transmission of a respiratory virus [8,9,18,19,20,21].

Airborne	Anything in the Air
Aerosol	Suspension (carried along with air currents) of particles in a gas
Droplet	Liquid particle that can potentially carry pathogens
Droplet Nuclei	Small particle (diameter less than 5 µm) that are the result of the desiccation of larger droplets
Bioaerosol	Aerosol composed of fungi, bacteria, and other micro-organisms and biological matter usually ranging from 1 nm to 0.1 mm
Particulate Matter	The sum of chemical and biogenic compounds, of natural and/or anthropogenic origin, whose size vary between 1 nm and 100 μm, and which are found in the air and can be diffused and transported even over long distance
Aerosol Transmission	Transmission of a pathogen either through large particles of respiratory fluids (droplets), or through smaller particles that can remain aerosolized (droplet nuclei). This transmission mode can occur over larger distances, and does not require close contact between the susceptible and infected individuals
Droplet Transmission	Short range, direct transmission of a pathogen over short distances (<3 m) through large droplets (diameter upper 5 µm) whose trajectories are dictated by gravitational settling

**Table 2:** Nomenclature list of different abbreviations and parameters used in the formulas and models described in this work.

Code and Nomenclature		Unit	References
*P*	probability of infection	−	[32]
*N*	number of occupants in the room	−	[16,26,33]
*Ni*	number of infectors	−	[16,26,33]
*q*	quantum generation rate	h^−1^	[29,34]
*C_q_*	concentration of infectious quanta in the exhaled air	m^−3^	[34]
*Q*	pulmonary ventilation rate (breathing rate)	m^3^/h	[29,34]
*R_S_*	fraction of infectious particles penetrating through the mask of a susceptible individual	−	[16,26,33]
*R_I_*	fraction of infectious particles penetrating through the mask of an infector (infectious individual)	−	[16,26,33]
*ε*	risk factor	−	[16,26]
*s_r_*	transmissibility factor	−	[16,26]
*V*	volume of the room	m^3^	
*η*	mask filtration efficiency	−	[33]
*λ*	particle loss rate	h^−1^	[16,26]
*λ_a_*	ventilation rate	h^−1^	[16,26]
*λ_v_*	viral deactivation rate	h^−1^	[16,26]
*λ_s_*	particle sedimentation rate	h^−1^	[16,26]
*λ_f_*	air filtration rate	h^−1^	[16,26]
*t*	exposure time	H	[32]
*k*	concentration of CO_2_ in the exhaled air	ppm	[16,26]

**Table 3:** Maximum allowed CO_2_ level in terms of infection risk for different scenarios considered [16,34,38].

Scenario	Exposure Time *t* (h)	Mask Wearing *R_S_, R_I_*	Breathing Flow Rate *Q* (m^3^/h)	Concentration of Infectious Quanta *Cq* (m^−3^)	Excess CO_2_ Level Δ (ppm)
Classroom (teacher is the infector)	1.5	*R_S_* = 0.15 *R_I_* = 1	1.6	100	106
Classroom (student is the infector)	1.5	*R_S_* = 0.15 *R_I_* = 0.15	0.3	5	75,000
Indoor sport activity (no masks)	1	*R_S_* = 1 *R_I_* = 1	3.0	300	4
Meeting (with masks)	1	*R_S_* = 0.15 *R_I_* = 0.15	0.3	10	56,300
Meeting (no masks)	1	*R_S_* = 1 *R_I_* = 1	0.3	10	1267

*R_S_* and *R_I_* represent the fraction of infectious particles penetrating through the masks of the susceptible and infected population, respectively.

**Table 4:** Possible measures mitigating the risk of infection and their seasonal influence, efficacy, feasibility, and acceptability. ID of the considered measures, efficiency, feasibility, and acceptability rated by a panel of experts and potential seasonal influence on the measures.

Continuous Measures					
*Factor Influencing Airborne Transmission*	*Mitigation Measures*	*Seasonal Influence on the Measures*	*Efficacy*	*Feasibility*	*Acceptability*
Ventilation	(1) Room ventilation (doors and windows)	Yes	+++	+++	++
	(2) Room ventilation (HVAC systems)	No	+++	++	+++
Viral concentration	(3) Portable air cleaners	No	++	++	+++
	(4) Filters within fixed HVAC systems	No	++	+	+++
	(5) Air quality monitoring	No	++	+++	+++
	(6) External UV-C lighting	No	+	+	+
	(7) Mask usage	No	+++	+++	++
Room occupancy	(8) Reducing occupants	No	+++	++	++
	(9) Reducing time	No	++	++	+
Temperature and humidity	(10) Temperature and humidity control (HVAC)	Yes	+	+	++
**Measures Prior to Room Occupancy**					
*Factor Influencing Virus Transmission*	*Mitigation Measures*	*Seasonal Influence on the Measures*	*Efficacy*	*Feasibility*	*Acceptability*
Number of infectors	(11) Refusing unvaccinated individuals	No	+	+	+
	(12) Body temperature control	No	+	+	+
	(13) Refusing symptomatic individuals	Yes	++	++	++
	(14) Self-testing before access	No	++	+	+
	(15) Presentation of COVID-19 certificate	No	+	++	+

UV-C, short-wave ultraviolet; HVAC, heating, ventilation, and air conditioning. Efficacy, feasibility and acceptability were assessed using the following scale: + (low), ++ (middle) and +++ (high).

**Table 5:** Summary of the ranking of 15 proposed mitigation measures (top and last three).

Most Efficient	Most Feasible	Most Acceptable
Ventilation	Ventilation (doors and windows)	Ventilation (mechanical)
Mask wearing	Mask wearing	Air filters
Reducing room occupancy	Air quality monitoring	Air quality monitoring
**Least Efficient**	**Least Feasible**	**Least Acceptable**
External UV-C lighting	External UV-C lighting	External UV-C lighting
T and RH control	T and RH control	Reducing occupation time
Refusing access to certain individuals	Refusing access to certain individuals	Refusing access to certain individuals

UV-C—Short-wave UV; T and RH—temperature and relative humidity.

## Data Availability

Not applicable.

## Appendix A

PRISMA 2020 Checklist.

**Section and Topic**	**Item #**	**Checklist Item**	**Location Where Item Is Reported**
**TITLE**			
Title	1	Identify the report as a systematic review.	1
**ABSTRACT**			
Abstract	2	See the PRISMA 2020 for Abstracts checklist.	2
**INTRODUCTION**			
Rationale	3	Describe the rationale for the review in the context of existing knowledge.	2–4
Objectives	4	Provide an explicit statement of the objective(s) or question(s) the review addresses.	4
**METHODS**			
Eligibility criteria	5	Specify the inclusion and exclusion criteria for the review and how studies were grouped for the syntheses.	4–5
Information sources	6	Specify all databases, registers, websites, organizations, reference lists, and other sources searched or consulted to identify studies. Specify the date when each source was last searched or consulted.	4–5
Search strategy	7	Present the full search strategies for all databases, registers, and websites, including any filters and limits used.	4–5
Selection process	8	Specify the methods used to decide whether a study met the inclusion criteria of the review, including how many reviewers screened each record and each report retrieved, whether they worked independently, and if applicable, details of automation tools used in the process.	4–5
Data collection process	9	Specify the methods used to collect data from reports, including how many reviewers collected data from each report, whether they worked independently, any processes for obtaining or confirming data from study investigators, and if applicable, details of automation tools used in the process.	4–5
Data items	10a	List and define all outcomes for which data were sought. Specify whether all results that were compatible with each outcome domain in each study were sought (e.g., for all measures, time points, and analyses), and if not, the methods used to decide which results to collect.	5–6
	10b	List and define all other variables for which data were sought (e.g., participant and intervention characteristics, and funding sources). Describe any assumptions made about any missing or unclear information.	8
Study risk of bias assessment	11	Specify the methods used to assess risk of bias in the included studies, including details of the tool(s) used, how many reviewers assessed each study, and whether they worked independently, and if applicable, details of automation tools used in the process.	6
Effect measures	12	Specify for each outcome the effect measure(s) (e.g., risk ratio and mean difference) used in the synthesis or presentation of results.	8 (Table 2)
Synthesis methods	13a	Describe the processes used to decide which studies were eligible for each synthesis (e.g., tabulating the study intervention characteristics and comparing against the planned groups for each synthesis (item #5)).	5–6
	13b	Describe any methods required to prepare the data for presentation or synthesis, such as handling of missing summary statistics, or data conversions.	Not appropriate
	13c	Describe any methods used to tabulate or visually display results of individual studies and syntheses.	8 (Table 2)
	13d	Describe any methods used to synthesize results and provide a rationale for the choice(s). If meta-analysis was performed, describe the model(s), method(s) to identify the presence and extent of statistical heterogeneity, and software package(s) used.	5–6 No meta-analysis
	13e	Describe any methods used to explore possible causes of heterogeneity among study results (e.g., subgroup analysis and meta-regression).	Not appropriate
	13f	Describe any sensitivity analyses conducted to assess robustness of the synthesized results.	No appropriate
Reporting bias assessment	14	Describe any methods used to assess risk of bias due to missing results in a synthesis (arising from reporting biases).	6–20
Certainty assessment	15	Describe any methods used to assess certainty (or confidence) in the body of evidence for an outcome.	6–20
**RESULTS**			
Study selection	16a	Describe the results of the search and selection process, from the number of records identified in the search to the number of studies included in the review, ideally using a flow diagram.	4–6
	16b	Cite studies that might appear to meet the inclusion criteria, but which were excluded, and explain why they were excluded.	4–6
Study characteristics	17	Cite each included study and present its characteristics.	6 Appendix B
Risk of bias in studies	18	Present assessments of risk of bias for each included study.	6–20
Results of individual studies	19	For all outcomes, present for each study: (a) summary statistics for each group (where appropriate), and (b) an effect estimate and its precision (e.g., confidence/credible interval), ideally using structured tables or plots.	5–9
Results of syntheses	20a	For each synthesis, briefly summarize the characteristics and risk of bias among contributing studies.	6–20
	20b	Present results of all statistical syntheses conducted. If meta-analysis was done, present for each the summary estimate and its precision (e.g., confidence/credible interval) and measures of statistical heterogeneity. If comparing groups, describe the direction of the effect.	6–20 No meta-analysis
	20c	Present results of all investigations of possible causes of heterogeneity among study results.	6–20
	20d	Present results of all sensitivity analyses conducted to assess the robustness of the synthesized results.	Not appropriate
Reporting biases	21	Present assessments of risk of bias due to missing results (arising from reporting biases) for each synthesis assessed.	Not appropriate
Certainty of evidence	22	Present assessments of certainty (or confidence) in the body of evidence for each outcome assessed.	6–20
**DISCUSSION**			
Discussion	23a	Provide a general interpretation of the results in the context of other evidence.	6–20
	23b	Discuss any limitations of the evidence included in the review.	6–20
	23c	Discuss any limitations of the review processes used.	6–20
	23d	Discuss implications of the results for practice, policy, and future research.	15–20
**OTHER INFORMATION**			-
Registration and protocol	24a	Provide registration information for the review, including register name and registration number, or state that the review was not registered.	Not registered
	24b	Indicate where the review protocol can be accessed, or state that a protocol was not prepared.	−
	24c	Describe and explain any amendments to information provided at registration or in the protocol.	−
Support	25	Describe sources of financial or non-financial support for the review, and the role of the funders or sponsors in the review.	21
Competing interests	26	Declare any competing interests of review authors.	21
Availability of data, code, and other materials	27	Report which of the following are publicly available and where they can be found template data collection forms; data extracted from included studies; data used for all analyses; analytic code; any other materials used in the review.	21

## Appendix B

List of the retained publications for this systematic review with main characteristics.

**Reference (Number)**	**Author (Year)**	**Parameter(s) Described ***
1	WHO (2021)	*−*
2	WHO (2022)	*−*
3	Thanh Le et al. (2020)	*−*
4	So et al. (2020)	*−*
5	Mathieu et al. (2021)	*−*
6	Moriyama et al. (2020)	*T*, *RH*
7	Kohanski et al. (2020)	*λ*
8	Wang et al. (2021)	*P*, *q*, *Q*, *C_q_*, *N*, *N_i_*
9	Tang et al. (2021)	*η*
10	Chirico et al. (2020)	*T*, *RH*, *λ*
11	da Silva et al. (2021)	*T*, *RH*
12	Burridge et al. (2021)	Δ, *N*, *N_i_*, *λ*, *q*, *Q*, *C_q_*, *k*
13	Jones et al. (2020)	*λ*, *V*, *N*, *N_i_*, *Q*, *η*
14	Lelieveld et al. (2020)	*λ*, *q*, *Q*, *C_q_*, *V*, *η*
15	Azuma et al. (2020)	*T*, *RH*, *P*, *q*, *Q*, *C_q_*, *N*, *N_i_*, *λ*
16	Bazant et al. (2021)	*λ*, *P*, *q*, *Q*, *C_q_*, *N*, *N_i_*, *η*
17	Stabile et al. (2021)	Δ, *P*, *N*, *N_i_*, *λ*, *q*, *Q*, *C_q_*, *k*
18	Xie et al. (2021)	*−*
19	Santurtún et al. (2021)	*−*
20	Aganovic et al. (2021)	*T*, *RH*, *N*, *N_i_*, *λ*, *q*, *Q*, *C_q_*
21	Smieszek et al. (2019)	*P*, *λ*
22	Page et al. (2021)	*−*
23	Chatterjee et al. (2021)	*T*, *RH*
24	Netz et al. (2020)	*T*, *RH*, *λ*
25	Srinivasan et al. (2021)	*T*, *RH*, *λ*
26	Bazant et al. (2021)	Δ, *P*, *N*, *N_i_*, *λ*, *q*, *Q*, *C_q_*, *k*, *ε*, *η*
27	Delikhoon et al. (2021)	*T*, *RH*, *λ*
28	Pal et al. (2021)	*T*, *RH*
29	Coleman et al. (2021)	*q*, *Q*, *C_q_*
30	Trancossi et al. (2021)	*q*, *Q*, *C_q_*, *T*, *RH*, *λ*
31	Spena et al. (2020)	*T*, *RH*
32	Riley et al. (1978)	*T*, *RH*, *λ*
33	Shen et al. (2021)	*λ*, *P*, *q*, *Q*, *C_q_*, *N*, *N_i_*, *η*
34	Buonanno et al. (2020)	*q*, *Q*, *C_q_*, *λ*, *P*, *T*, *RH*
35	Dai et al. (2020)	*λ*, *P*, *q*, *Q*, *C_q_*, *N*, *N_i_*, *η*
36	Miller et al. (2021)	*q*, *Q*, *C_q_*, *λ*
37	Kurnitski et al. (2021)	*λ*, *P*, *q*, *Q*, *C_q_*, *N*, *N_i_*, *η*, *V*
38	Shen et al. (2021)	*λ*, *P*, *q*, *Q*, *C_q_*, *N*, *N_i_*, *η*, *V*
39	Beggs et al. (2021)	*T*, *RH*
40	Quraishi et al. (2020)	*T*, *RH*
41	Biryukov et al. (2020)	*T*, *RH*
42	Elsaid et al. (2021)	*T*, *RH*, *λ*
43	Bu et al. (2021)	*T*, *RH*, *λ*, *P*
44	Vassella et al. (2021)	Δ, *λ*, *k*, *T*, *RH*
45	Peng et al. (2021)	Δ, *λ*, *k*, *ε*, *P*
46	Vouriot et al. (2021)	Δ, *λ*, *k*, *q*, *Q*, *C_q_*, *P*
47	Chillon et al. (2021)	Δ, *λ*, *T*, *RH*
48	Lepore et al. (2021)	Δ, *λ*
49	Morawska et al. (2020)	*λ*
50	Lung et al. (2021)	*λ*
51	Lee et al. (2021)	*λ*, *V*, *ε*
52	Aguilar et al. (2021)	Δ, *λ*, *T*, *RH*, *V*
53	Rodriguez et al. (2021)	*λ*
54	Bono et al. (2021)	*λ*
55	Garcia de Abajo et al. (2020)	*λ*
56	ASHRAE (2019)	*−*
57	Melikov et al. (2020)	*λ*, *N*, *N_i_*, *V*
58	Park et al. (2021)	*λ*, *P*
59	Rencken et al. (2021)	*λ*, *Q*, *η*
60	Singer et al. (2022)	*λ*, *Q*
61	Ascione et al. (2021)	*λ*, *T*, *RH*
62	Duill et al. (2021)	Δ, *λ*, *Q*, *V*
63	Gil-Baez et al. (2021)	Δ, *λ*
64	Kulo et al. (2021)	Δ, *λ*, *T*, *RH*
65	Nazarenko et al. (2020)	*λ*
66	de Almeida et al. (2020)	*λ*
67	de Almeida et al. (2021)	*λ*
68	Lendvay et al. (2022)	*λ*
69	Kwon et al. (2021)	*T*, *RH*
* The nomenclature list of different abbreviations of parameters are described in Table 2.		

## Appendix C

Percentage of opening of doors and windows for natural ventilation in classrooms, offices, hallways, restaurants, libraries, and other university rooms within the University of Liège, and average temperature recorded.

This figure shows the amount of times the doors or windows of rooms within the University of Liège were open during at least 50% of the occupation time, reported by the occupants of said rooms. This was recorded during a period ranging from the 37nd week of 2021 to the 3rd week of 2022, excluding weeks 51– and 52–2021, as students are on study break and personnel occupation is scares due to holidays during these two weeks. The temperature was recorded at Liege-Bierset weather station and sourced on www.infoclimat.fr (accessed on 15 April 2022).
